# The Biology of Lactoferrin, an Iron-Binding Protein That Can Help Defend Against Viruses and Bacteria

**DOI:** 10.3389/fimmu.2020.01221

**Published:** 2020-05-28

**Authors:** Douglas B. Kell, Eugene L. Heyden, Etheresia Pretorius

**Affiliations:** ^1^Department of Biochemistry, Faculty of Health and Life Sciences, Institute of Integrative Biology, University of Liverpool, Liverpool, United Kingdom; ^2^The Novo Nordisk Foundation Center for Biosustainability, Technical University of Denmark, Lyngby, Denmark; ^3^Department of Physiological Sciences, Faculty of Science, Stellenbosch University, Stellenbosch, South Africa; ^4^Retired, Spokane, WA, United States

**Keywords:** lactoferrin, coronaviruses, iron, membrane receptors, HSPGs

## Abstract

Lactoferrin is a nutrient classically found in mammalian milk. It binds iron and is transferred via a variety of receptors into and between cells, serum, bile, and cerebrospinal fluid. It has important immunological properties, and is both antibacterial and antiviral. In particular, there is evidence that it can bind to at least some of the receptors used by coronaviruses and thereby block their entry. Of importance are Heparan Sulfate Proteoglycans (HSPGs) and the host receptor angiotensin-converting enzyme 2 (ACE2), as based on other activities lactoferrin might prevent severe acute respiratory syndrome coronavirus 2 (SARS-CoV-2) from attaching to the host cells. Lactoferrin (and more specifically enteric-coated LF because of increased bioavailability) may consequently be of preventive and therapeutic value during the present COVID-19 pandemic.

## Introduction

Lactoferrin (LF) or lactotransferrin has recently come under the spotlight, particularly with regards to the new coronavirus pandemic that started in 2019 (COVID-19). Diet and supplements support a well-functioning immune system, and favorably influence the body's ability to fight infection. Although LF is produced by the body itself, as a secretion by exocrine glands (such as maternal milk or tears) and secondary granules of human neutrophils (1), it can also be taken as a supplement, where it then acts as nutraceutical or functional food. Our particular focus is on its role as an oral supplement. Here we also collate some of the evidence that shows how LF may be an important nutrient to support host immunity, including as an antibacterial and antiviral agent, but particularly with the current COVID-19 pandemic in mind.

**Figure 1:**
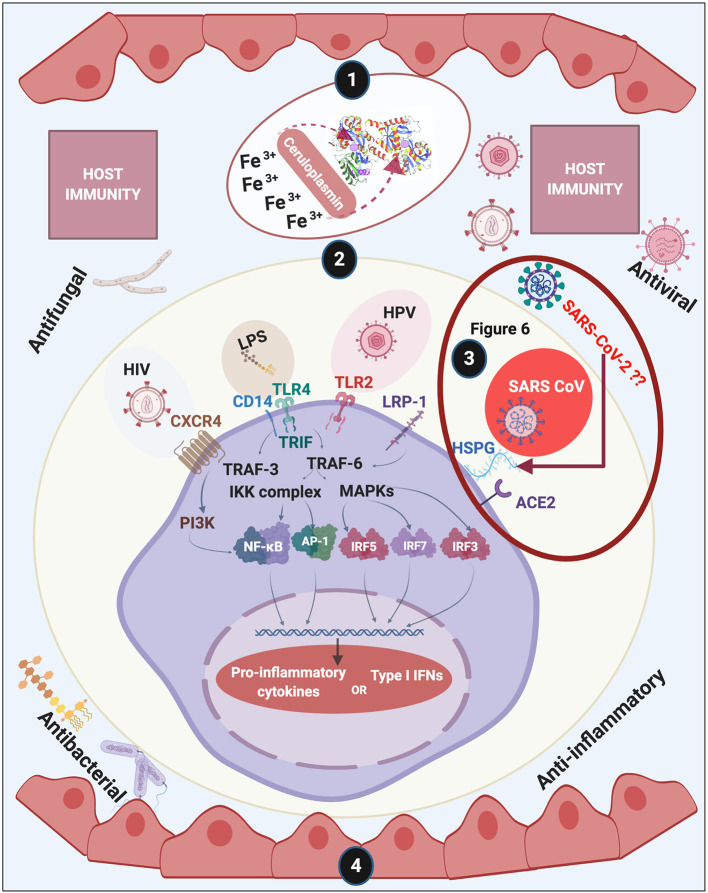
Overview of this review of lactoferrin (LF). We discuss (1) discovery and structure of LF; (2) LF membrane receptors and some of the bacteria, their products and viruses that might also bind to these receptors, (3) including how acute respiratory syndrome coronavirus 2 (SARS-CoV-2) (causing COVID-19) may interact with host cells (see Figure 6 and Conclusion for a detailed discussion); (4) and how LF assists with host immunity. Diagram created with BioRender (www.biorender.com).

We summarize what is already known about LF, including its immunological properties, as well as its antibacterial and antiviral activities. We also discuss how LF uses Heparan Sulfate Proteoglycans (HSPGs) on cell surfaces to facilitate entry. This is of particular importance to coronaviruses, as these viruses are considered to bind to the host cell by attaching first to HSPGs using them as preliminary docking sites on the host cell surface. LF is known to interfere with some of the receptors used by coronaviruses, it may thus contribute usefully to the prevention and treatment of SARS CoV-2 infections. In COVID-19 infection, LF may therefore have a role to play, not only sequestering iron and inflammatory molecules that are severely increased during the cytokine burst, but also possibly in assisting by occupying receptors and HSPGs. LF might also prevent virus accumulation by the host cell, as well as rolling activity and entering of the virus via the host receptor angiotensin-converting enzyme 2 (ACE2). It has been 20 years since the discovery of ACE2, and since its discovery it has been found to be expressed in numerous tissues, including the lungs and the cardiovascular system (2). During 2020, there has been a renewed interest in this receptor, due to the interactions of novel coronaviruses and their interactions with ACE2 (3–5). South and co-workers in 2020 also investigated whether ACE2 blockade is a suitable option to attenuate COVID-19 (5). The use of recombinant human ACE2 (rhACE2) as ACE receptor competitor for binding has also been investigated (6, 7). There is also interest in the therapeutic targeting of HSPGs, and Hondermarck and co-workers suggested that is seems an easy way to inhibit SARS-Cov-2 infectivity (8). Here we also suggest that LF might be used as both a preventive and therapeutic supplement in the COVID-19 pandemic, by preventing interactions between the virus and both HSPGs and possibly ACE2. We summarize the layout of this paper in Figure 1.

## Discovery and Structure

**Figure 2:**
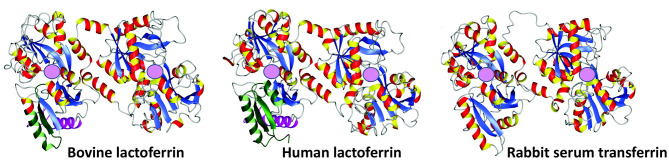
Crystal structures of bovine lactoferrin (PDB code = 1BLF), human lactoferrin (1B0L), and rabbit serum transferrin (1JNF). Adapted from Vogel (10). Pink spheres represent ferric iron (Fe^3+^) binding sites.

Human LF is a cationic glycosylated protein consisting of 691 amino acids (9) folded into two globular lobes (80 kDa bi-lobal glycoprotein) (10), that are connected by an α-helix (11, 12). Bovine LF contains 689 amino acids (13). LF was first discovered and isolated from bovine milk in 1939 (14), and is a member of the transferrin family (60% amino acid sequence identity with serum transferrin) (11). LF and transferrin have similar amino acid compositions, secondary structures (including their disulphide linkages), and tertiary structures, although they differ in terms of biological functions (11, 15, 16) (see Figure 2). There are also three different isoforms: LF-α is the iron-binding isoform, while LF- β and LF-g both have ribonuclease activity but do not bind iron (11, 17). When it is iron-rich it is referred to hololactoferrin and when iron-free apolactoferrin (18). The tertiary structures of the two forms are significantly different: apolactoferrin is characterized by an open conformation of the N-lobe and a closed conformation of the C-lobe, while both lobes are closed in the hololactoferrin (18). Human LF and bovine LF possess high sequence homology and have very similar antibacterial, antifungal, antiviral, antiparasitic, anti-inflammatory, and immunomodulatory activities (19–21). Consequently, it is common to give the bovine form rather than say a recombinant human form as a supplement. Bovine LF is also deemed a “generally recognized as safe” substance by the Food and Drug Administration (FDA, USA), and is commercially available in large quantities (19).

Due to its similarities to transferrin, which is the main iron transporting molecule in serum (22, 23), α-LF possesses iron binding capabilities (24, 25), and it can chelate two ferric irons (Fe^3+^) (26). LF binds one ferric iron atom in each of its two lobes; however, an important attribute is that it does not release its iron, even at pH 3.5. This is of importance as this property assures iron sequestration in infected tissues where the pH is commonly acidic (27). In the context of its iron-binding capabilities, it means that when it binds ferric and siderophore-bound iron, it limits the availability of essential iron to microbes (27).

In healthy individuals, iron is largely intracellular and sequestered within ferritin or as a co-factor of cytochromes and FeS proteins, and as haem complexed to hemoglobin within erythrocytes. Circulating iron is rapidly bound by transferrin (28, 29). When erythrocytes lyse and hemoglobin or haem is released into the circulation, their hemoglobin is captured by haptoglobin, and haem by hemopexin (30). Here, circulating serum ferroxidase ceruloplasmin is of importance, as LF can bind to ceruloplasmin, such that a direct transfer of ferric iron between the two proteins is possible (31). A direct transfer of ferric iron from ceruloplasmin to lactoferrin prevents both the formation of potentially toxic hydroxyl radicals (32) and the utilization of iron by pathogenic bacteria. LF is therefore an important player in preventing bacteria from acquiring and sequestering iron, which [with the possible exception of *Borrelia burgdorferi* (33)]; they require for growth and virulence. LF also acts as biomarker, as it is commonly upregulated when the host is suffering from various kinds of disease. See Table 1 for selected references.

**Table 1:** Lactoferrin as a major player in host defense and iron binding, and its use as biomarker for various diseases.

**Area of action**	**References**
Protecting neonates via breast milk	(34–41)
LF in cervicovaginal mucosa and female reproductive tract; antibacterial, antifungal antiparasitic, antiviral	(42–45)
LF in the airways	(46, 47)
Mucosal surfaces, allergen-induces skin infections	(48)
Neutrophil extracellular trap (NET) production	(49)
Saliva and its antimicrobial activities and iron binding	(50–52)
Saliva as biomarker for neurological diseases	(53–55)
Saliva as biomarker for periodontal disease and oral dryness	(56–59)

## Lactoferrin and Its Membrane Receptors

LF is thought to exert its main biological activities following interaction with receptors on target cells. There are in fact many LF receptors, though sometimes one is referred to as “the” lactoferrin receptor. They have been detected in multiple tissues and cell types including intestinal epithelial cells and lymphocytes (60, 61). Receptors that bind LF include CD14 (62), LDL receptor-related protein-1 (LRP-1/CD91) (63–65) intelectin-1 (omentin-1) (66), Toll-like receptor 2 and 4 (TLR4) (67) and cytokine receptor 4 (CXCR4) (68) (see Table 2). Importantly, LF also binds to heparan sulfate proteoglycans (HSPGs), which are cell-surface and extracellular matrix macromolecules that are composed of a core protein decorated with covalently linked glycosaminoglycan (GAG) chains (86, 87, 98, 99). See Table 2. Different receptors express at vastly different levels in different tissues; thus intelectin-1 is really expressed only in the intestine (https://www.proteinatlas.org/ENSG00000179914-ITLN1/tissue), while LRP1 is far more widely distributed https://www.proteinatlas.org/ENSG00000123384-LRP1/tissue. These multiple receptors arguably underpin the substantial and widespread effects that LF can induce, since only when multiple targets are hit simultaneously can one normally have major effects (103, 104).

**Table 2:** Receptors for lactoferrin, cells where these receptors are present, and other molecules and/or components that might bind to these receptors.

**Receptor for lactoferrin**	**Cell types where receptor are present**	**Selected references**
Lactoferrin receptor/LRP-1/CD91/apoE receptor or the chyclomicron remnant receptor	Multiple tissues and cell types including intestinal epithelial cell lymphocytes, fibroblasts, neurons, hepatocytes, endothelial cells	(62) (60, 69–71)
Intelectin-1 (omentin-1)	Visceral (omental and epicardial) fat, mesothelial cells, vascular cells, airway goblet cells, small intestine, colon, ovary, and plasma	(66, 72)
TLR2 and TLR4	Endothelial cells, platelets, neutrophils	(73–80)
CXCR4	Platelets, endothelial cells, neutrophils, T-cells	(78, 81–83)
CD14	Macrophages, neutrophils	(62, 84, 85)
Heparan sulfate proteoglycans (HSPGs),	Epithelial cells, endothelial cells, fibroblasts, lymphocytes	(86, 87)
Interleukin 1	Various cells	
**Selected molecules and entities that bind to these receptors, other than lactoferrin**		
**Receptor**	**Molecule or cellular entity**	**References**
Lactoferrin receptor	Bacteria	(30)
LRP-1	Amyloid beta (Aβ)	(69, 88–90)
Intelectin-1 (omentin-1)	Microbial sugars, including β-D-galactofuranose (β-Galf), D-glycerol 1-phosphate, d-*glycero*-D-*talo*-oct-2-ulosonic acid (KO), and 3-deoxy-d-*manno*-oct-2-ulosonic acid (KDO)	(91)
TLR4	Bacterial lipopolysaccharides (LPSs) Herpex simplex	(78, 92–94)
CXCR4	Viruses (including HIV)	(78, 95, 96)
CD14	LPS, H7N9 Influenza virus	(92, 97)
Heparan sulfate proteoglycans (HSPGs)	Various viruses, including HIV and SARS-CoV	(86, 87, 98–102)

The entry of bacteria, bacterial products or viruses into host cells may also occur via some of these receptors. Such binding evokes signaling systems and pathways involving, amongst others, mitogen-activated protein kinase (MAPK) (105), NF-κB (106), activator protein 1 (AP-1) (107), and various interferon regulatory factors (IRFs) [for a comprehensive review see (108)]. During infection, activation of these signaling pathways results in a cellular response that shares multiple cytoplasmic components, leading ultimately to the activation of a complex biomolecular network. Phosphorylation of relevant substrates (e.g., enzymes, microtubules, histones, and transcription factors) plays a crucial role in determining the host's cellular response (109). Viruses (110, 111), as well as bacteria (112), interact with and bind to HSPGs, using this proteoglycan as entry into the cell (see also Figure 1). LF acts as an important element in host defense mechanisms by binding to these receptors, but also binding to HSPG on cells, since these are locations where binding to bacteria and their cell wall products as well as viruses occur. The membrane-penetrating peptide HIV-tat, released from HIV-infected cells, also enters surrounding cells using HSPGs (86, 98). This binding capacity allows LF to compete with such molecules for receptor occupancy (113, 114), and therefore plays a vital role in host immunity (20). LF can also serve to prevent nephrotoxicity, e.g., of cisplatin (115).

## Lactoferrin Transport

Small molecules, including pharmaceutical drugs, require solute carriers of the SLC family (116) to effect their uptake (117–124). Lactoferrin, as a protein, is far too large to exploit such a route, and instead passes from the stomach via epithelial cells and into the blood using endocytosis (125, 126), especially via Peyer's patches (127), and when it is encapsulated (“enterically formulated”) in liposomes (128–130). This uptake then occurs mostly via the lymphatic rather than the portal circulation (131, 132). LF can also enter, and be reabsorbed from, the bile (125). Blood LF can further be transported to the CNS via cerebrospinal fluid (133, 134) and via the Blood Brain Barrier (63, 133).

## Lactoferrin: an Important Element in Host Defense

### Neutrophils and Lactoferrin

**Figure 3:**
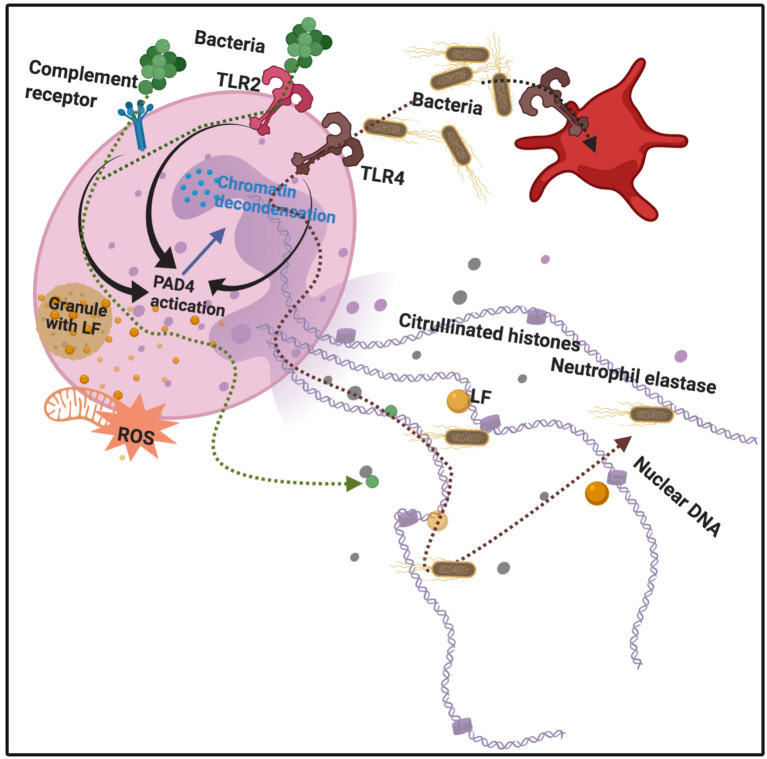
Bacterial binding to various receptors, e.g., Toll-like receptors 2 and 4 (TLR2 and 4), as well as complement receptors, leads to protein arginine deiminase 4 (PAD4) activation, followed by chromatin decondensation, hypercitrullination of histones 3 and 4 in the nucleus, and nuclear membrane disruption. Granules also release lactoferrin. Neutrophil Extracellular Traps (NETs) and their protein constituents (including lactoferrin) are released from the neutrophil. Adapted from Jorch and Kubes (142) and Law and Gray (143). Bacteria are expelled and trapped in the NETs. Diagram created with BioRender (https://biorender.com/).

LF plays an important role in host defense, upon its release from the neutrophil (26). LF also enhances natural killer cell activity in immune defense (135) and can restrict the entry of the virus into host cells during infection. As part of the host's inflammatory response, leucocytes, including neutrophils, release LF from their granules, where it is normally stored. Activated neutrophils also release chromatin fibers, known as neutrophil extracellular traps (NETs), which trap and kill, amongst others, bacteria (1, 136). These NETs likewise modulate both acute and chronic inflammation (137, 138). NETs are also found in various autoimmune conditions such as rheumatoid arthritis, systemic lupus erythematosus (139, 140). Interestingly, 10^6^ human neutrophils can release 15 μg of LF (26). In addition to DNA and histones, NET fibers contain extranuclear proteins and proteins such as elastase, myeloperoxidase (MPO), and LF (141). LF may also serve as an intrinsic inhibitor of NETs release into the circulation, and may therefore be central in controlling NETs release (1). See Figure 3.

### Bacteria and Lactoferrin

**Figure 4:**
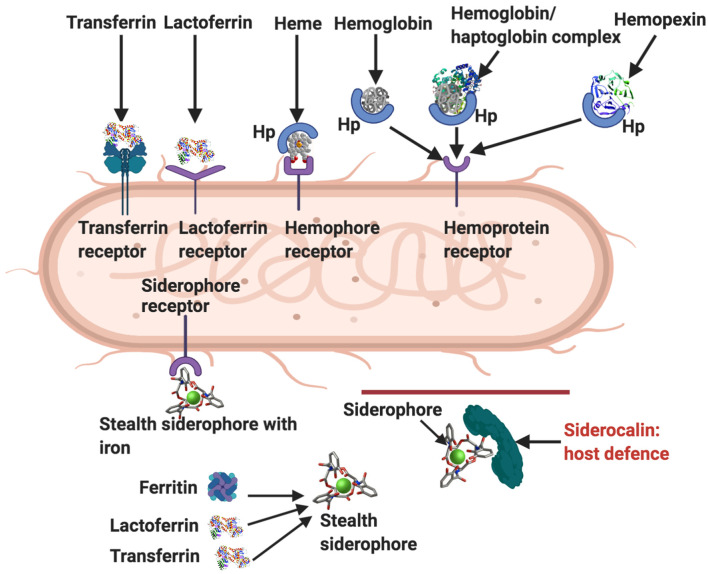
Ways by which bacteria acquire iron [adapted from (19, 30)]. Transferrin receptor, lactoferrin receptor, hemophore (Hp), hemophore receptor, and hemopexin. Siderophores remove iron from lactoferrin, ferritin and transferrin, and also from the environment. Stealth siderophores are modified in such a way as to prevent siderocalin binding. A primary bacterial defense against siderocalin involves the production of stealth siderophores. Modified from Rosa et al. and Skaar (19, 30). Diagram created with BioRender (https://biorender.com/).

One of the most well-known characteristics of LF is that it is antibacterial (19, 144–148), antiviral (99, 149–151), antifungal (152–154), anti-inflammatory (26), and anti-carcinogenic (155). Its ability to of limit iron availability to microbes is one of its crucial amicrobial properties. Bacteria have, however, developed various ways to sequester iron (156). Figure 4 shows how bacteria acquire iron through receptor-mediated recognition of transferrin, hemopexin, hemoglobin, or hemoglobin-haptoglobin complexes and also LF (30). As well as binding it directly from the environment, bacterial siderophores can obtain iron by removing it from transferrin, lactoferrin, or ferritin (32). These siderophore-iron complexes are then recognized by receptors on the bacterium (30). Host innate immune functions are supported by the circulating protein, siderocalin, also known as Neutrophil gelatinase-associated lipocalin (NGAL), lipocalin2 or Lcn2 as it inhibits siderophore-mediated iron acquisition and release (30).

Although LF has various means to counteract bacteria as part of its immune function (131), it is also capable of being hijacked to benefit the activities of bacteria. Thus, bacteria can also exploit LF by removing its bound ferric iron (19, 30). This process involves (1) synthesis of high-affinity ferric ion chelators by bacteria, (2) iron acquisition through LF or transferrin binding, mediated by bacterial-specific surface bacterial receptors, (3) or iron acquisition through bacterial reductases, which are able to reduce ferric to ferrous ions (19, 144–148).

Several Gram-negative pathogens including members of the genera *Neisseria* and *Moraxella* have evolved two-component systems that can extract iron from the host LF and transferrin (157). *N. meningitidis* is a principal cause of bacterial meningitis in children. While the majority of pathogenic bacteria employ siderophores to chelate and scavenge iron (158), *Neisseria* has evolved a series of protein transporters that directly hijack iron sequestered in host transferrin, lactoferrin, and hemoglobin (159). The system consists of a membrane-bound transporter that extracts and transports iron across the outer membrane (TbpA for transferrin and LbpA for lactoferrin), and a lipoprotein that delivers iron-loaded lactoferrin/transferrin to the transporter (TbpB for transferrin and LbpB for lactoferrin) (157). LbpB binds the N-lobe of lactoferrin, whereas TbpB binds the C-lobe of transferrin (157). However, more than 90% of LF in human milk is in the form of apolactoferrin (160), which competes with siderophilic bacteria for ferric iron, and disrupts the proliferation of these microbial and other pathogens. Similarly LF supplements may play an important role to counteract bacterial processes. LF is consequently a significant element of host defense (19), and its levels may vary in health and during disease. It is hence known to be a modulator of innate and adaptive immune responses (161).

### Viruses and Lactoferrin

LF has strong antiviral activity against a broad spectrum of both naked and enveloped DNA and RNA viruses (99, 149–151). LF inhibits the entry of viral particles into host cells, either by direct attachment to the viral particles or by blocking their cellular receptors (discussed in previous paragraphs) (149). Some of the viruses that LF prevents from entering host cells e.g., *Herpes simplex* virus (162), human papillomavirus (163), human immunodeficiency virus (HIV) (164), and rotavirus (165). These viruses typically utilize common molecules on the cell membrane to facilitate their invasion into cells, including HSPGs (Figure 1). HSPGs provide the first anchoring sites on the host cell surface, and help the virus make primary contact with these cells (99, 162). HSPGs can be either membrane bound, or in secretory vesicles and in the extracellular matrix (86). It has been shown that LF is able to prevent the internalization of some viruses by binding to HSPGs (86).

### COVID-19 and Lactoferrin

COVID-19 is caused by severe acute respiratory syndrome coronavirus 2 (SARS-CoV-2). Many COVID-19 patients develop acute respiratory distress syndrome (ARDS), which leads to pulmonary edema and lung failure, and have liver, heart, and kidney damages. These symptoms are associated with a cytokine storm (166, 167) manifesting elevated serum levels of interleukin (IL) IL-1β, IL-2, IL-7, IL-8, IL-9, IL-10, IL-17, granulocyte colony-stimulating factor (G-CSF), Granulocyte-Macrophage Colony Stimulating Factor (GM-CSF), interferon (IFN)γ, tumor necrosis factor (TNF)α, Interferon gamma-induced protein 10 (IP10), Monocyte Chemoattractant Protein-1 (MCP1), macrophage inflammatory protein 1(MIP1)A and MIP1B (168). IL-22, in collaboration with IL-17 and TNFα, induces antimicrobial peptides in the mucosal organs. IL-22 also upregulates mucins, fibrinogen, anti-apoptotic proteins, serum amyloid A, and LPS binding protein (169); therefore, IL-22 may contribute to the formation of life-threatening oedema with mucins and fibrin (170), seen in SARS-CoV-22 and SARS-CoV patients (168).

The 2003 SARS-CoV strain, that also causes severe acute respiratory syndrome, attaches to host cells via host receptor ACE2 (171). This type I integral membrane protein receptor is a well-known receptor for respiratory viruses, and is abundantly expressed in tissues lining the respiratory tract (111). During COVID-19 infection, SARS-CoV-2 also enters host cells via the ACE2 receptor (172). ACE2 is highly expressed on human lung alveolar epithelial cells, enterocytes of the small intestine, and the brush border of the proximal tubular cells of the kidney (99). HSPGs are also one of the preliminary docking sites on the host cell surface and play an important role in the process of SARS-CoV cell entry (99). There is no current confirmed information that SARS-CoV-2 binds to HSPGs, however, LF blocks the infection of SARS-CoV by binding to HSPGs (99). It is not presently known whether LF binds to ACE2, but it does bind to HSPGs (99). Whether SARS-CoV-2 also enters host cells via HPSGs in the same way, as does (the 2003) SARS-CoV clearly warrants further investigation.

**Figure 5:**
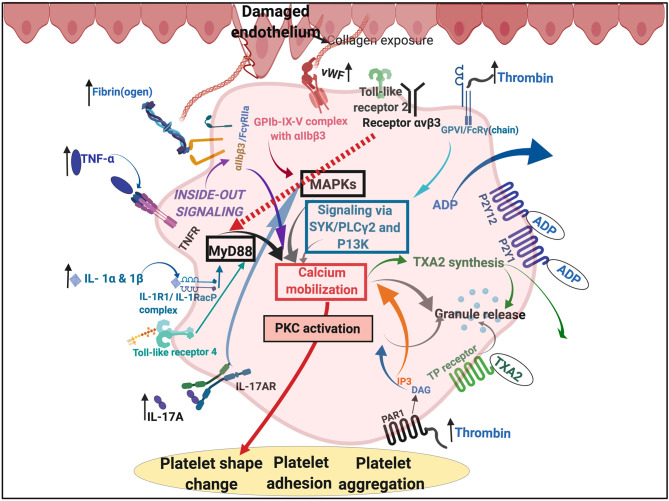
Simplified platelet signaling and receptor activation during disease with main dysregulated molecules thrombin, fibrin(ogen), von Willebrand Factor (vWF) interleukins (IL) like IL-1α, IL-1β, and IL17A and cytokines like TNF-α. Diagram created with BioRender (https://biorender.com/).

Of particular interest, and in the context of this paper, is the set of interactions between SARS-CoV-2 and host platelets. This is of importance, as COVID-19 infection, can cause hyperinflammation due to a cytokine storm (166). Pathogens like the influenza virus and *Francisella tularensis*, do trigger life-threatening cytokine storms (173). Such a cytokine storm will significantly affect platelets, as platelets have many receptors where these inflammatory molecules may bind (173) (see Figure 5). Circulating cytokines and inflammagens will hyperactivate platelets, causing low platelet count (thrombocytopenia), and a significant chance of hypercoagulation. Thrombocytopenia is associated with increased risk of severe disease and mortality in patients with COVID-19, and thus serves as clinical indicator of worsening illness during hospitalization (174, 175). Patients with type 2 diabetes are also particularly prone to increased levels of circulating inflammatory cytokines and hypercoagulation (76). COVID-19 patients without other comorbidities but with diabetes are at higher risk of severe pneumonia, excessive uncontrolled inflammatory responses and a hypercoagulable state (176). Guo and co-workers in 2020 also found that serum levels of IL-6, C-reactive protein, serum ferritin, and D-dimer, were significantly higher in diabetic patients compared with those without, suggesting that patients with diabetes are more susceptible to an inflammatory storm eventually leading to rapid deterioration of the patient with COVID-19 (140). Acute pulmonary embolism has also been reported in COVID-19 infection (177). Focal accumulation of activated platelets within the oedematous area *ex vivo* correlated well with the size of the pulmonary embolism (178). Interestingly, anticoagulant therapy, mainly with (intravenous) heparin (and mainly with low molecular weight heparin, LMWH), appears to be associated with better prognosis in severe COVID-19 patients (179).

In COVID-19 infection, LF may have a role to play in not only sequestering iron and inflammatory molecules that are severely increased during the cytokine burst, but also possibly in assisting in occupying receptors and HSPGs to prevent virus binding. Receptor occupancy is an important characteristic of LF, when taken as supplement. Furthermore, it may assist in preventing thrombocytopenia, and hypercoagulation, both prominent features of COVID-19 infection.

## Lactoferrin as a Nutraceutical

There is little doubt that oral LF can be of health benefit to the host, and while it is not considered to be absolutely necessary for mammalian life (so it is not a vitamin), it is reasonable to class it as a nutraceutical along with a variety of other molecules such as those mentioned in various papers (180, 181). As a nutraceutical, the bioavailability of LF would clearly be an important consideration in its use for the prevention or treatment of COVID-19. Enteric coating of LF capsules has been proposed as a measure to maximize the uptake of LF by the receptors located in the brush-border of the small intestine (182). Enteric coating allows LF release some distance from LF-degrading pepsin activities in the stomach, allowing it to remain intact, in the form capable of binding small intestinal LF receptors for uptake and eventual transfer into the systemic circulation (182). In a rodent study, the “absorption” of enteric-formulated LF was approximately 10-fold higher than that of regular LF introduced into the stomach of experimental animals (128). In view of these investigations, the authors of this paper regard enteric-coated LF as superior to regular LF supplements with respect to bioavailability and potential application for the prevention or therapy for coronaviruses such as the SARS-Cov-2 involved in COVID-19.

### Nutritional Sources, Availability and Uses for Lactoferrin as Supplement

There is considerable LF availability in various forms and sources. Table 3 shows some of the sources and the references to research where it has been used to treat various conditions.

**Table 3:** Lactoferrin sources as supplements, and examples where it has been used to treat various conditions.

**Lactoferrin sources as supplements**	
**Product**	**References**
Bovine and human milk	Morinaga Industries in Japan (183) DoMO Food Ingredients, a subsidiary of Friesland Dairy Foods, in the Netherlands (184)
Human recombinant lactoferrin	Talactoferrin from Agennix, Inc., Houston, Texas, USA (184)
Lactoferrin expression in transgenic rice	Ventrus Biosciences, New York City, New York, USA (184)
Transgenic cattle expressing human lactoferrin	(185, 186)
Transgenic maize	Meristem therapeutics, Clermont-Ferrand, France (184)
**Lactoferrin supplementation in treatment of various diseases**	
Might be useful in treating sepsis or necrotizing enterocolitis in preterm neonates	(184)
Support for vaginal health	(187)
LF may play a protective role in host defense against SARS-CoV infection through binding to HSPGs and blocking the preliminary interaction between SARS-CoV and host cells (cell culture study)	(99)
LF is a modulator of innate immune responses in the urinary tract and has potential application in novel therapeutic design for urinary tract infection (animal study)	(188)
Possible therapy against *Candida albicans* in the oral cavity (a hypothesis)	(189)
Protection against *Chlamydia trachomatis* (cell culture study)	(190)
Treatment of taste and smell abnormalities after chemotherapy	(52)
LF supplements and food with high levels of LF for oral health	(99, 191)
LF treatment of black stain associated with of iron metabolism disorders with lactoferrin	(192)
Aerosolized bovine LF counteracts infection, inflammation and iron dysbalance in a cystic fibrosis mouse model of *Pseudomonas aeruginosa* chronic lung infection	(193)
LF inhalations for lung health	(194)
LF for optimal skin moisture	(195)

## Conclusions

**Figure 6:**
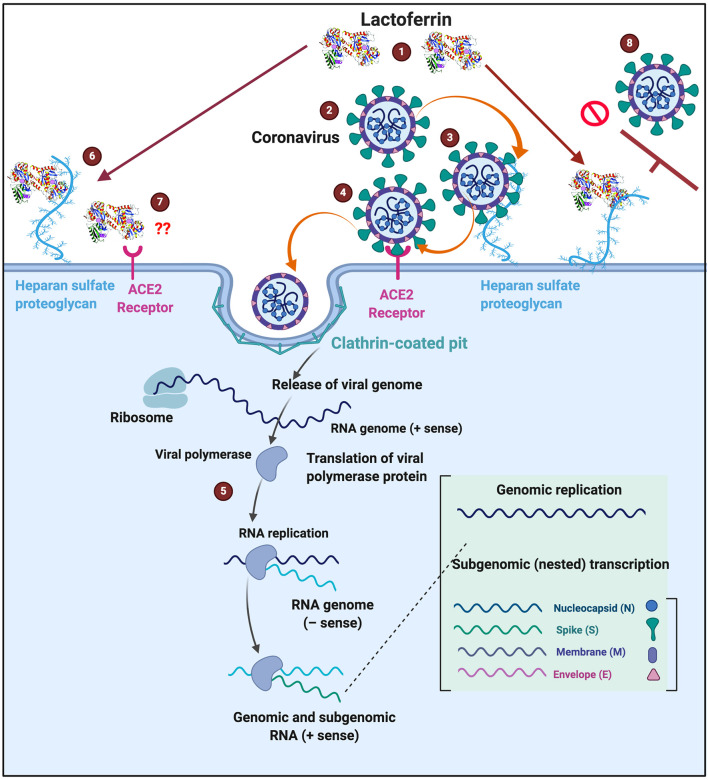
Possible action of (1) lactoferrin by occupying binding sites of (2) SARS-CoV-2 that causes COVID-19. (3) Entry into host cells occur when SARS-CoV-2 first attaches to Heparan sulfate proteoglycans (HSPGs). This attachment initiates the first contact between the cell and the virus, concentrating the virus on the cell surface, (4) followed attaching of the virus to the host receptor (ACE2) and association and entering are then facilitated via clathrin-coated pits (5) Virus replication can then happen inside the cell. (6) One of the characteristics of Lactoferrin, is that it attaches to HSPGs. (7) Currently we do not know if ACE2 is also a receptor for lactoferrin. (8) Lactoferrin may block the entry of SARS-CoV-2 into the host cell, by occupying HPSGs, thereby preventing SARS-CoV-2 initial attachment and accumulation on the host cell membrane. COVID-19 infection template adjusted from www.biorender.com.

Lactoferrin clearly has immunological benefits, as well as having an important antibacterial and antiviral role. Because it is known to interfere with some of the receptors used by coronaviruses, it may contribute usefully to the prevention and treatment of coronavirus infections. Figure 6 shows a possible scheme on how LF might interfere with SARS-CoV-2 binding. The binding of LF to HSPGs prevents the first contact between virus and host cells and thus prevents subsequent infection (99). HSPGs themselves are not sufficient for SARS-CoV entry. However, in SARS-CoV infections, the HSPGs play an important role in the process of cell entry (99). The anchoring sites provided by HSPGs permit initial contact between the virus and host cells and the concentration of virus particles on cell surface. SARS-CoV bound to HSPGs then rolls onto the cell membrane and scans for specific entry receptors, which leads to subsequent cell entry (99). LF enhances natural killer cell activity and stimulates neutrophil aggregation and adhesion in immune defense (135) and can restrict the entry of the virus into host cells during infection. We suggest that this process might be the same for COVID-19 (see Figure 6 for a visual representation), thereby offering useful strategies for prevention and treatment. Currently, there is also a renewed interest in ACE2 and HSPG blocking, as discussed in the introduction (5–8). LF may therefore be an excellent supplement to take, not only as a contribution to prevention but perhaps as a therapy in the event COVID-19 is diagnosed.

## Data Availability Statement

The original contributions presented in the study are included in the article/supplementary material, further inquiries can be directed to the corresponding author/s.

## Author's Note

Another recent review (196) has also highlighted the potential utility of lactoferrin as an antiviral.

## Author Contributions

EP wrote the paper. DK edited and wrote part of the paper. EH provided clinical input and edited the paper. All authors approved submission of the paper.

## Conflict of Interest

The authors declare that the research was conducted in the absence of any commercial or financial relationships that could be construed as a potential conflict of interest.

## Abbreviations

LF: Lactoferrin; lactotransferrin
SARS-CoV: acute respiratory syndrome coronavirus
LRP-1/CD91: LDL receptor-related protein-1
TLR2 and 4: Toll-like receptor 2 and 4
CXCR4: cytokine receptor 4
GAG: glycosaminoglycan
AP-1: activator protein 1
NF-κB: NF-kappa beta
IRF: Interferon regulatory factor
MAPK: Mitogen-activated protein kinase
HSPG: Heparan sulfate proteoglycans
ACE2: Angiotensin-converting enzyme 2
IL: Interleukin
G-CSF: Granulocyte colony-stimulating factor
GM-CSF: Granulocyte-Macrophage Colony Stimulating Factor
IFN: Interferon
TNFα: Tumor necrosis factor alpha
IP10: Interferon gamma-induced protein 10
MCP1: Monocyte Chemoattractant Protein-1
(MIP1) A and B: Macrophage inflammatory protein 1 (A and B)
LMWH: Low molecular weight heparin
vWF: von Willebrand Factor
PAD4: protein arginine deiminase 4
NETS: Neutrophil extracellular traps.
